# Factors associated with uptake of voluntary medical male circumcision, Mazowe District, Zimbabwe, 2014

**DOI:** 10.11604/pamj.2014.19.337.5245

**Published:** 2014-11-28

**Authors:** Maxwell Rupfutse, Cremence Tshuma, Mufuta Tshimanga, Notion Gombe, Donewell Bangure, Maureen Wellington

**Affiliations:** 1 Department of Community Medicine, University of Zimbabwe, Harare 00263, Zimbabwe; 2 Ministry of Health and Child Care Mashonaland Central Province Medical Directorate, Zimbabwe

**Keywords:** Male circumcision, HIV, Mazowe district, Zimbabwe

## Abstract

**Introduction:**

Voluntary Medical Male Circumcision (VMMC) is the surgical removal of the foreskin by a trained health worker. VMMC was introduced in Zimbabwe in 2009. It is of concern that the programme performance has been below expectations nationally and in Mazowe district. Zimbabwe is unlikely to meet its 2015 target of circumcising 1 200 000 men aged between 15 and 29 years and unlikely to enjoy maximum benefits of VMMC which include prevention of HIV, sexually transmitted infections and cervical cancer. We therefore broadly aimed at identifying factors influencing the level of VMMC uptake in Mazowe district.

**Methods:**

An analytic cross-sectional study was carried out in Mazowe district. A multi-stage probability sampling strategy was used to select 300 men aged between 18 and 49 years. Pretested interviewer administered questionnaires, key informant interviews and focus group discussions were used to collect data. Quantitative data was analysed using Epi info where odds ratios and p-values were calculated. Qualitative data was analysed thematically.

**Results:**

Being of Shona origin (AOR= 7.69 (95%CI 1.78-33.20)), fear of pain (AOR= 7.09 (95%CI 2.58-19.47)) and fear of poor wound healing (AOR= 2.68 (95%CI 1.01-7.08)) were independently associated with being uncircumcised while having a circumcised friend and encouragement by a friend or relative were independently associated with being circumcised.

**Conclusion:**

Fear of pain, fear of poor wound healing and encouragement by a friend or relative were associated with circumcision status. Widening use of surgical devices and third part referrals may assist in scaling up the programme.

## Introduction

The estimated number of people infected with the Human Immunodeficiency Virus (HIV) in 2012 was 35.3 million and 23,5million of these were in Sub-Saharan Africa [1]. Of the 35.3 million people infected with HIV in 2012 globally, 30.7 million were adults aged above 15 years and 13.3 million of these were men [1]. There were 2.3 million new HIV infections globally in 2012 and 1.6 million HIV related deaths [1]. In Zimbabwe, the prevalence of HIV stood at 15% according to the Zimbabwe Demographic Health Survey (ZDHS) of 2010-2011 [2]. The same survey indicated that the prevalence of HIV among women and men aged between 15-49 years was 18% and 12% respectively [2]. In Mashonaland Central Province, 13.7% of people aged between 15-49 years were infected with HIV (15.1% in women and 12.3% in men) [2].

Several strategies have been implemented in order to curb the spread of HIV including Voluntary Medical Male Circumcision (VMMC). VMMC is defined as the surgical removal of the foreskin by a trained health worker [3]. It is a strategy to prevent the spread of HIV that was recommended by the World Health Organization (WHO) in 2007 [4]. It was specifically recommended in countries with high HIV prevalence and low prevalence of male circumcision such as Zimbabwe [4]. This followed results of Randomized Control Trials (RCTs) done in South Africa, Uganda and Kenya which demonstrated that VMMC reduces HIV transmission by up to 60% [5]. It has also been demonstrated that circumcising 80% of men could prevent 45% of new HIV infections between the years 2011 and 2015 [6]. However, it is worth noting that VMMC offers partial protection to HIV [7]. This calls for use of other HIV prevention methods in conjunction with the strategy. Moreover, concerns have been raised about possibility of compensatory disinhibition after the procedure [7].

Apart from the partial prevention of HIV transmission, VMMC has been found to have other medical benefits. These include improvement of personal hygiene, reduction of sexually transmitted infections such as genital herpes, syphilis and Chlamydia, prevention of penile cancer, prevention of ballanitis, prevention of paraphymosis, reduction in risk of urinary tract infections and reduction of cervical cancer risk in partners of circumcised men [8].

VMMC was seen to be cost effective. An initial investment of US1, 5billion dollars between 2011 and 2015 to achieve 80% coverage of VMMC services in 14 priority countries in Southern and Eastern Africa countries and a further investment of US0, 5billion dollars from 2016 to 2025 to maintain that coverage of 80% would result in net savings of US16, 5 billion dollars between 2011 and 2025 [9].

Mashonaland Central Province is one of the ten provinces of Zimbabwe. It is bordered by Mashonaland East Province to the east and south, Mashonaland West Province to the west, Mozambique and Zambia to the north. The provincial town is Bindura, which is located about 87 kilometres north east of Harare. The total population of Mashonaland Central Province according to the 2012 census is 1 152 520 people comprised of 567 140 males and 585 380 females [10]. There are eight administrative districts in the province. These are Bindura, Shamva, Mazowe, Guruve, Mbire, Centenary, Mount Darwin and Rushinga. Mazowe District is the most populous district in the province with a total of 243 999 people (21% of provincial population) [10].

Voluntary Medical Male Circumcision (VMMC) was introduced in Zimbabwe in 2009 with the aim of circumcising 1 200 000 men between 15 and 29 years by the year 2015. The circumcision is done at static or outreach sites. Static sites are mainly district and provincial hospitals while outreach services are provided at clinics. A lot of effort and resources have been invested into the VMMC programme. These include mass media campaigns, social mobilizations in schools and communities, school holiday campaigns and incentives to health workers. The programme has also made use of popular musicians and local leaders. It is of concern that despite these efforts, by the end of 2012, three years after the start of the program, only 91 335 (7.6%) men had received VMMC surgery. At this rate, Zimbabwe is unlikely to meet its target and unlikely to enjoy maximum benefits of VMMC which include prevention of HIV, cervical cancer and penile cancer. In Mashonaland central province, four districts are offering VMMC services under Population Services International (PSI). According to monthly return forms for the period 1/04/2013 to 30/09/2013, the combined total for the four districts did not reach the monthly target of 800 and 1350 during school holiday campaigns (April and August). The study was carried out to identify factors influencing the level of VMMC uptake in Mazowe district.

## Methods

An analytic cross-sectional study was carried out at Mazowe District, Zimbabwe. Men aged between 18 and 49 years, living in Mazowe district, medically circumcised or not and gave written consent was enrolled into the study.

Using 26% of participants who cited fear of pain as a reason for unwillingness to get circumcised in a study by Mavhu W et al on prevalence and factors associated with knowledge of and willingness for male circumcision in rural Zimbabwe, at 95% confidence interval and using n= z^2^p(1-p)/d^2^,a minimum sample size of 296 participants was enrolled into the study (Where n = sample size, z = maximum allowable error risk = 1.96, p = proportion of participants who cited fear of pain as a reason for unwillingness to get circumcised = 26% and d = standard error (difference between point estimate and population parameter) = 0.05).

A multi-stage probability sampling strategy was used. Selection of study participants was household based. Seven wards out of the thirty-five wards in Mazowe district were randomly selected using computer generated random numbers. The wards in Mazowe District were numbered from one to thirty-five and the wards which correspond to the computer generated numbers were selected. Two villages from each ward were then selected using a similar procedure. A total of 22 participants were interviewed in six of the villages and 21 participants were interviewed in eight of the villages. Three wards with the highest number of villages contributed the six villages with 22 participants as shown in table 3 below. The village head's homestead was our reference point. The homestead nearest to the village head's homestead was then selected as a source for the first participant of each village. One willing man aged between 18 and 49 years was then randomly selected using the lottery method. Names of eligible and willing men present at the time of visit were written on a piece of paper and thrown in a box from which one was blindly picked. The individual whose name was picked became the study participant for that homestead. The next nearest homestead was then selected until a total of 22 or 21 interviews were held. If there was no willing and eligible man at any homestead, the interviewer would skip to the next homestead.

Permission to conduct the study was sought from the Provincial Medical Director (PMD) for Mashonaland Central Province, the District Medical Officer (DMO) for Mazowe District, the Health Studies Office (HSO), the Joint Parirenyatwa Hospital and College of Health Sceinces Reaserch Ethics Committeee (JREC) and the Medical Research Council of Zimbabwe (MRCZ).

Interviewer administered questionnaires were used to collect data on demographic, socio-economic, personal, environmental and programmatic factors associated with uptake of VMMC. The researcher administered the questionnaires. The study participants were followed up at their homes with assistance from local village health workers and environmental health technicians. Interviews were carried out at the participants’ homes. Key informant interviews were done to determine challenges the VMMC programme is facing in Mazowe District and what can be done to scale up the programme. Review of hospital/clinic records were done to confirm circumcision details of medically circumcised men, that is, when and where were they circumcised. Three Focus Group Discussions (FGD) were done with eighteen village heads in Mazowe District to explore cultural issues that influence uptake of VMMC. The village heads were drawn from three different wards (wards 4, 7 and 19). The researcher used village heads’ or community meetings to assemble the village heads. An interviewer guide was used to guide the discussions. Each focus group was consisted of six village heads of similar age, a moderator and a recorder. The discussions were held in Shona and were recorded on an audio tape. They were then summarised, transcribed and translated on the same day.

Data was analysed using Epi Info version 3.5.1 to generate frequencies, proportions, p-values and odds ratios with their 95% confidence intervals. Stratified analysis was done to check for confounding factors and effect modifiers such as ethnicity and marital status, and logistic regression was done to identify independent factors that are associated with VMMC uptake.

## Results

A total of 300 participants were enrolled into the study. More than two thirds of the respondents were married and 72% of the respondents had at least secondary education. Ninety-two percent of the respondents were Shona and eight percent were Nyanja. The prevalence of circumcision was 12.3% among the Shona and 50% among the Nyanja. The circumcised and uncircumcised had similar demographic characteristics except for ethnicity. Table 1 shows the socio-demographic characteristics and circumcision status of the respondents.

**Table 1:** Socio-demographic characteristics and circumcision status of respondents, Mazowe district, Zimbabwe, 2014

Variable	Frequency n (%)
**Marital status**	
Married	216 (72.0)
Single	84 (28.0)
**Education level**	
None	8 (2.7)
Primary	75 (25.0)
Secondary	207 (69.0)
Tertiary	10 (3.3)
**Religion**	
Orthodox	56 (18.7)
Apostolic	64 (21.3)
Pentecostal	54 (18.0)
Muslim	5 (1.7)
Traditional	17 (5.7)
None	104 (34.7)
**Ethnicity**	
Shona	276 (92.0)
Nyanja	24 (8.0)
**Employment Status**	
Formally employed	47 (15.7)
Informally employed	43 (14.3)
Not employed	210 (70.0)
**Circumcision status**	
Uncircumcised	254 (87.7)
Circumcised	46 (15.3)
Median age in years 30 (Q1 = 24,Q3 = 36)	

Twenty-four percent of the circumcised respondents were circumcised traditionally. However, their circumcision status was not confirmed. The circumcision status of those circumcised at clinics/hospitals was confirmed by use of medical records. Eight of the medically circumcised respondents developed complications (seven had excessive swelling and only one had excessive pain). More than a third did not abstain from sexual activities for the recommended six weeks.

Reasons for not getting circumcised given by the uncircumcised respondents were fear of pain (42.1%), long abstinence period (30.3%), being too old for circumcision (14.9%), possibility of partner infidelity during the abstinence period (6.3%), being HIV positive (5.5%) and fear of reduced sexual performance post circumcision (4.3%). Four (1.6%) uncircumcised respondents said that they did not want to be circumcised as they had heard about people dying during traditional circumcision ceremonies. One hundred and seventeen (46.1%) of the uncircumcised respondents did not have a reason for not getting circumcised or were just procrastinating.

VMMC was reported to be appropriate and acceptable by all respondents of Nyanja origin where circumcision is practised culturally as a right to passage and is done at puberty. About 16.1% of the uncircumcised respondents and 6.5% of the circumcised respondents were not satisfied by services they receive at clinics or hospitals in Mazowe district. Table 2 shows facilitators and barriers to VMMC uptake. The reasons given by those not satisfied with services received at clinics/hospitals were rude nurses (52.3%), long queues or long waiting time at clinics or hospitals (31.8%) and shortage of medicines at health facilities (25.0%).

**Table 2:** Facilitators and barriers to vmmc uptake, Mazowe district, Zimbabwe, 2014

Variable	Uncircumcised n (%)	Circumcised n (%)
**Distance to clinic/hospital**		
< 5 km	150 (59.1)	30 (65.2)
> 5 km	104 (40.9)	16 (34.8)
**Satisfied with clinic/hospital services**		
Yes	213 (83.9)	43 (93.5)
No	41 (16.1)	3 (6.5)
**Partner influence on circumcision uptake**		
Not influential	96 (41.6)	14 (31.1)
Influential	74 (32.0)	12 (26.7)
Very influential	61 (26.4)	19 (42.2)
**Appropriateness of VMMC in Shona culture**		
Inappropriate	71 (28.0)	3 (6.5)
Neutral	85 (33.5)	15 (32.6)
Appropriate	98 (38.6)	28 (60.9)
Acceptability of VMMC in Shona culture		
Unacceptable	45 (17.7)	2 (4.3)
Neutral	80 (31.5)	11 (23.9)
Acceptable	129 (50.8)	33 (71.7)

The Shona were seven times more likely to be uncircumcised compared to the Nyanja. Fear of pain was eleven times more common among the uncircumcised compared to the circumcised. Those who fear knowing their HIV status were three times more likely to be uncircumcised compared to those who do not fear knowing their HIV status. Those with a circumcised friend were more likely to be circumcised compared to those without a circumcised friend. Other significant factors associated with VMMC uptake were fear of poor wound healing, having a circumcised relative, encouragement by a friend or relative and discussing circumcision with female partner. Table 3 shows factors associated with VMMC uptake.

**Table 3:** Factors associated with vmmc uptake, Mazowe district, Zimbabwe, 2014

Variable	Uncircumcised n (%)	Circumcised n (%)	OR (95% CI)
**Ethnicity**			
Shona	242 (95.3)	34 (73.9)	7.12
Nyanja	12 (4.7)	12 (26.1)	(3.25-11.34)
**Fear of pain**			
Yes	190 (74.8)	10 (21.7)	11.03
No	64 (25.2)	36 (78.3)	(5.18-23.52)
**Fear of bleeding**			
Yes	63 (24.8)	7 (15.2)	1.84
No	191 (75.2)	39 (84.8)	(0.78-4.32)
**Fear of poor wound healing**			
Yes	197 (77.6)	18 (39.1)	5.38
No	57 (22.4)	28 (60.9)	(2.78-10.42)
**Fear of knowing HIV status**			
Yes	95 (37.4)	7 (15.2)	3.33
No	159 (62.6)	39 (84.8)	(1.43-7.74)
**Circumcised friend**			
Yes	92 (36.2)	39 (84.8)	0.10
No	162 (63.8)	7 (15.2)	(0.04-0.24)
**Circumcised relative**			
Yes	78 (30.7)	36 (78.3)	0.12
No	176 (69.3)	10 (21.7)	(0.06-0.26)
**Encouraged by relative/friend**			
Yes	82 (32.3)	39 (84.8)	0.086
No	172 (67.7)	7 (15.2)	(0.04-0.20)
**Discussed circumcision with partner**			
Yes	107 (46.3)	29 (65.9)	0.45
No	124 (53.7)	15 (34.1)	(0.23-0.88)

The association between circumcision status and fear of poor wound healing was not modified nor confounded by marital status. The association between discussing circumcision with partner and circumcision status was confounded by marital status. There was no effect modification nor confounding by ethnicity of the association between circumcision status and having a circumcised relative. The association between circumcision status and having a circumcised friend was not modified nor confounded by ethnicity.

On multivariate analysis, being of Shona origin, fear of pain and fear of poor wound healing were independently associated with being uncircumcised while having a circumcised friend and encouragement by a friend or relative were independently associated with being circumcised. Table 4 shows the independent factors associated with VMMC. The frequency of condom use was significantly higher among the circumcised compared to the uncircumcised as shown in Table 5. There was no significant difference in the number of sexual partners in the previous three months. On rating the knowledge levels for both the uncircumcised and the circumcised, 29.7% were poor, 49.3% were average and 21% were good (0-1 = poor, 2-3 = average, 4-5 = good). Table 6 shows respondents’ knowledge on VMMC.

**Table 4:** Independent factors associated with vmmc uptake, Mazowe district, Zimbabwe, 2014

Variable	AOR	95% CI
Ethnicity (Shona)	7.70	1.78-33.20
Fear of pain	7.09	2.58-19.47
Fear of poor wound healing	2.68	1.01-7.08
Circumcised friend	0.19	0.06-0.65
Encouraged by someone	0.21	0.07-0.67

**Table 5:** Sexual activities and hiv testing, Mazowe district, 2014

Variable	Uncircumcised n (%)	Circumcised n (%)	P-value
**Sexually active**			0.077^*^
Yes	222 (87.4)	44 (95.7)	
No	32 (12.6)	2 (4.3)	
**Used condom last sex**			0.016
Yes	69 (31.2)	22 (50.0)	
No	153 (68.8)	22 (50.0)	
**Number of sexual partners in last three months**			0.700
Less than 2	169 (76.1)	35 (79.5)	
2 or more	53 (23.9)	9 (20.5)	
**Ever tested for HIV**			<0.001
Yes	95 (37.4)	40 (87.0)	
No	159 (62.6)	6 (13.0)	

* Fisher exact

### Results of Focus Group Discussions (FGDs) on VMMC, Mazowe district, 2014

Focus group discussions were held with three groups of six village heads each (a total of eighteen village heads). All the village heads expressed some knowledge on VMMC in Mazowe district. They mentioned prevention of HIV, sexually transmitted diseases and cervical cancer in female partners as benefits of VMMC. They also knew that VMMC was being offered for free in the district. The challenges that were mentioned in all the three groups concerning VMMC included inappropriateness of male circumcision among the Shona who are mainly Christians since male circumcision is culturally and religiously practised by Muslims and/or Nyanja people. One village head said “they want to turn us into Muslims” with a strong conviction in his voice and expression. There were also conceptions or misconceptions of side effects of male circumcision such as excessive pain, excessive swelling, disfigurement of the male sexual organ, long abstinence period, reduction of sexual performance and pleasure and even death that were raised in all the three groups. Five village heads were concerned about possible behavioural disinhibition following VMMC. They were worried that circumcised people especially youths would think that they are now protected from HIV and that their sexual organ has been “sharpened” resulting in risky sexual activities. Village heads in all the three focus groups reported that they were not encouraging their people to be circumcised because of the above mentioned views but may be able to do so if provided with enough information regarding male circumcision side effects and assurance that VMMC is not driven by the Muslim religion.

### Results of Key Informant Interviews on VMMC, Mazowe district, 2014

The respondents for the key informant interviews were Government Medical Officers (GMO) (2), District Nursing Officer (DNO) (1), Provincial AIDS Coordinator (PAC) (1), District Health Promotion Officer (DHPO) (1) and VMMC nurses (2). It was the opinion of all the key informants that the VMMC programme in Mazowe district was not performing according to expectations. The reported challenges facing the programme in the district were lack of coordination and mobilisation of partners and stakeholders such as community leaders, school authorities and traditional healers. The suggested solutions to these challenges were strengthening awareness among stakeholders and sensitisation and mobilisation of community leadership on VMMC through provision of adequate information on the benefits of VMMC and addressing existing misconceptions about VMMC.

## Discussion

The prevalence of male circumcision established in this study is higher than the 10% that was reported by the WHO in 2009 [11]. This may suggest a positive effect of the VMMC programme in Mazowe district on the MC prevalence. The wide difference in circumcision prevalence between the Shona and the Nyanja can be explained by the fact that male circumcision is more acceptable and appropriate among the Nyanja and they traditionally circumcise. It is worth noting that circumcision status was based on respondents’ response. It was not confirmed by a physical examination.

The acceptability of male circumcision among the Shona was relatively low (50.4%). This finding is similar to the 52% reported by Mavhu et al in Mazowe in their study on prevalence and factors associated with knowledge of and willingness for male circumcision in rural Zimbabwe [12]. Focus group discussions reviewed that the acceptability of VMMC was hugely affected by cultural and/or religious beliefs. Bailey RC et al had similar findings [13]. They found that religious and cultural beliefs make VMMC difficulty among non-circumcising communities. This makes sensitisation and mobilisation of community leaders crucial for the success of the VMMC programme. Unlike the study done by Mavhu et al in Mazowe, this study did not find any association between age, marital status, level of education and circumcision uptake [12]. Mavhu et al established that age, marital status and level of education were significantly associated with circumcision status [12]. However, the two studies concurred that Nyanja were more likely to be medically circumcised compared to the Shona. The finding that fear of pain was associated with circumcision uptake should be taken with caution as the circumcised individuals had gone through the experience while the uncircumcised had not. However, Mavhu W et al had similar findings in Mazowe district [12]. They found that fear of pain was one on the reasons for circumcision unwillingness. The association between fear of poor wound healing and VMMC can be linked to the long abstinence period and fear of potential partner infidelity that were given as reasons for not being circumcised. This is further supported by the finding that more than a third of the circumcised individuals had abstinence periods of less than six weeks. Marya P et al had similar findings in Tanzania [14]. They noted that the long wound healing period resulting in abstinence for a prolonged period was a barrier to VMMC uptake. Thus development and use of surgical devices that shorten the healing period may increase VMMC uptake.

Fear of knowing HIV status coupled with the pre-requisite that only HIV negative men are circumcised is a bottleneck to the success of the programme. The HIV positivity rate of about 1% reported in the VMMC programme by PSI shows that it is mostly HIV negative people who go to VMMC clinics for possible circumcision. These men would most likely have been tested for HIV outside the VMMC programme making the success of the programme dependent on the performance of other HIV testing programmes. The performance of the VMMC programme can be improved by addressing this pre-requisite. The importance of third party referrals in marketing was further emphasised by the finding that those who were encouraged by someone were more likely to be circumcised. Use of circumcised friends and/or relatives in marketing the programme can assist in improving VMMC uptake. The study determined that female partners play an important role in men's decision to be circumcised or not. This is because male circumcision has a number of sexual implications such as the long abstinence period, fears of partner infidelity and perceptions regarding changes in sexual performance. Layer E H et al had similar findings in their study on the experiences of females in the circumcision of their male partners [15]. They established that females are important male circumcision influencers.

## Conclusion

The prevalence of male circumcision in Mazowe district was 15.3%. Ethnicity was an independent significant demographic factor associated with VMMC uptake. The significant independent socio-economic factors associated with VMMC uptake were having circumcised relative, having circumcised friend and having been encouraged by someone to undergo circumcision. Female partners were found to be important male circumcision influencers. The independent procedure related factors significantly associated with VMMC uptake were fear of pain and fear of poor wound healing. About 70% of respondents had average to good knowledge of VMMC.
